# Toward on-chip, in-cell recordings from cultured cardiomyocytes by arrays of gold mushroom-shaped microelectrodes

**DOI:** 10.3389/fneng.2012.00021

**Published:** 2012-08-24

**Authors:** Anna Fendyur, Micha E. Spira

**Affiliations:** The Alexander Silberman Life Sciences Institute and the Harvey M. Kruger Family Center for Nanoscience, The Hebrew University of JerusalemJerusalem, Israel

**Keywords:** cardiomyocyte electrophysiology, electroporation, multi-electrode array, field potential, action potential

## Abstract

Cardiological research greatly rely on the use of cultured primary cardiomyocytes (CMs). The prime methodology to assess CM network electrophysiology is based on the use of extracellular recordings by substrate-integrated planar Micro-Electrode Arrays (MEAs). Whereas this methodology permits simultaneous, long-term monitoring of the CM electrical activity, it limits the information to extracellular field potentials (FPs). The alternative method of intracellular action potentials (APs) recordings by sharp- or patch-microelectrodes is limited to a single cell at a time. Here, we began to merge the advantages of planar MEA and intracellular microelectrodes. To that end we cultured rat CM on micrometer size protruding gold mushroom-shaped microelectrode (gMμEs) arrays. Cultured CMs engulf the gMμE permitting FPs recordings from individual cells. Local electroporation of a CM converts the extracellular recording configuration to attenuated intracellular APs with shape and duration similar to those recorded intracellularly. The procedure enables to simultaneously record APs from an unlimited number of CMs. The electroporated membrane spontaneously recovers. This allows for repeated recordings from the same CM a number of times (>8) for over 10 days. The further development of CM-gMμE configuration opens up new venues for basic and applied biomedical research.

## Introduction

Multiunit, non-invasive extracellular recordings by substrate integrated planar Micro-Electrode Arrays (MEAs) is currently the prime electrophysiological methodology for long-term electrophysiological analysis of cultured cardiomyocyte (CM) activity. These MEAs serve as a platform for screening drugs, and elaborating therapeutic strategies (Sanchez-Bustamante et al., 2008; Yeung et al., 2009; Matsa et al., 2011; Law et al., 2012), the development of personalized medicine (Itzhaki et al., 2011) and may be instrumental in evaluating the use of cultured cells for functional integration with damaged heart tissue (Sekine et al., 2011). Similar planar MEAs are also used to interface cultured neurons or for brain-machine interfaces (Fromherz, 2006; Nicolelis and Lebedev, 2009; Jones et al., 2011). Monitoring CM electrical activity by planar MEA limits the electrophysiological information to extracellular field potentials (FPs) generated by action potentials (APs) in the vicinity of the electrodes. Detailed analysis of CM FP requires extensive computations, which rely on estimated parameters (Omura, 1970; Banach et al., 2003). On the other hand, the excellent signal-to-noise ratio provided by sharp-intracellular microelectrodes (Purves, 1981) and patch-electrodes (Sakmann and Neher, 1984) makes it possible to extract essential biophysical parameters underlying spike generation mechanisms and the properties of electrical synapses that couple different types of cardiac cells (Zipes and Jalife, 2009). Nevertheless, the use of sharp or patch microelectrodes is limited to a small number of cells, and because of continuous endogenous mechanical contraction, the friction between the rigid electrodes, and the cells leads to membrane rupture and cell death. In addition, patch-electrodes perfuse the cytoplasm and alter the intracellular composition of the cells thus limiting their use. To bypass the difficulties of using intracellular electrophysiological approaches to monitor electrical activity from contracting muscle cells, many laboratories now use voltage- or calcium-sensitive dyes (Herron et al., 2012). Although very effective for monitoring the beating rhythms, spread of APs and assessing the effects of drugs on these parameters, the loading of indicators may affect the electrophysiological properties of the cells (Nygren et al., 2003). Furthermore, imaging of the free intracellular calcium concentration kinetics reflects complex processes of calcium influx through voltage gated calcium channels, the release of calcium from intracellular stores, and the removal of calcium by a large number of mechanisms. Thus, currently the use of voltage sensitive dyes or calcium indicators cannot fully substitute for electrophysiological approaches.

To circumvent the limitations of planar-MEA, sharp- and patch-electrodes (as detailed above) and the side effects of various molecular probes, we began to explore in this study the use of micrometer size gold mushroom-shaped microelectrodes (gMμEs)-based MEA for intracellular recordings of APs from beating cultured rat CM.

**Figure 1:**
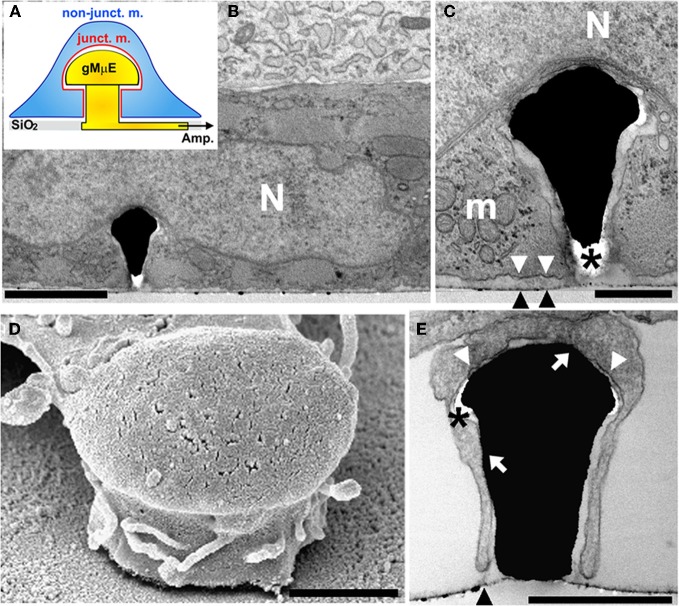
**A cultured cardiomyocyte engulfing a gMμE. (A)** Schematic representation of a CM (blue) engulfing a gMμE (yellow). Shown are the non-junctional membrane that faces the bathing solution (non-junct. m. in blue) and the junctional membrane that faces the gold mushroom shaped microelectrode (junct. m. in red). **(B)** A transmission electron-micrograph depicting a thin section through a CM engulfing a gMμE. The base of the electrode is not seen in the plane of the section. **(C)** Enlargement of the extracellular space interfacing the cardiomyocyte plasma membrane and the “cap” of the gMμE. **(D)** A scanning electron micrograph of a branch extending from a cultured cell partially engulfing a gMμE. **(E)** A transmission electron micrograph of a delicate cell extension engulfing a gMμE. N—nucleus, m—mitochondria, black arrow heads point to the culture substrate, white arrow heads point to the plasma membrane that faces the substrate, white arrows point to areas of close physical contact between the plasma membrane and the gMμE, * depicts post fixation formed “holes” in the embedding material. Calibrations: **B** = 2 μm, **C** = 0.5 μm, **D, E** = 1 μm.

In earlier studies from our laboratory we developed the use of gMμE-array to monitor intracellular synaptic and APs from cultured Aplysia neurons (Spira et al., 2007; Hai et al., 2009a,b, 2010a,b). The main principles underlying the neuron-gMμE interface were: the initiation of mechanisms by which cultured neurons actively engulf gMμEs that protrude from the surface of the device (see Figure 1A for schematic representation), the formation of high seal resistance between the plasma membrane and the gMμEs and the increased conductance of the plasma membrane facing the gMμE's cap (Hai et al., 2009a,b). Our studies were followed by three additional reports that used nanofabricated pillars and field effect transistors to gain intracellular recordings from cultured cells (Duan et al., 2012; Robinson et al., 2012; Xie et al., 2012).

We report here that when coated by laminin (Sanchez-Bustamante et al., 2008), the geometry of the gMμEs is sufficient to promote the engulfment of the protruding gMμEs by cultured CM (Figure 1), thus permitting gMμEs with a relatively small surface area (~10 μm^2^) to record endogenously generated FPs by individual myocytes for many days. In response to a short electroporation pulse delivered through the gMμEs, the functional CM-gMμE configuration is converted from an extracellular recording configuration to accessing intracellular recordings of attenuated APs. The shape and duration of the recorded APs is similar to those recorded intracellularly from rat CM. Autonomous recovery of the electropores generated at the plasma membrane facing the gMμEs spontaneously revert the recording mode to extracellular. The device enables repeated transition from the extracellular to the intracellular mode of recordings over a number of days despite the continuous beating of the CM.

## Methods

### gMμE-based MEA fabrication

Arrays of gMμE electrodes for electrical measurements were prepared on 200 μm thick glass wafers (AF45 Schott Glass) as previously described (Hai et al., 2009a, 2010b; Fendyur et al., 2011). Briefly, wafers were coated with a Ti (10 nm)/Au (100 nm) layer by way of evaporation, spin-coated with photoresist AZ-1505 (4000 RPM), and baked for 30 min at 90°C after which a first photolithographic process was performed to define the conduction lines by Au/Ti wet etch. Next a second lithographic step using S-1813G2 photoresist was performed to open holes for the deposition of the gMμE stalks as well as the contact pads. Next the gMμEs were formed by way of gold electroplating at a current density of 0.15 A/cm^2^ for 15–20 min. The photoresist layer was stripped off and a layer of silicon oxide (~3000 Angstrom) was deposited by chemical vapor deposition. This layer serves to passivate the conducting electrode lines. A third layer of photoresist was then applied. A third lithographic step was used to expose the contact pads and the caps of the gold mushrooms followed by wet oxide etch to selectively remove the oxide from the contact pads and the mushroom caps. Retrospective SEM of the gMμEs revealed that the oxide on the upper (third) part of the stalk was also etched. Wafers were then diced and underwent manual bonding to 62-pad printed circuit boards to which 21 mm glass rings were attached to create a recording bath chamber for the culturing medium.

### Fabrication of gold mushroom shaped micro protrusions matrices for electron microscopy

Scanning- and transmission-electron microscopic imaging were conducted using dissociated cultured CMs grown on matrices of gold-mushroom shaped protrusions (gMμP). The fabrication of gMμP matrices were prepared on 200 μm glass wafers (AF45 Schott Glass) by means of photolithography and electroplating techniques, as described above.

The slides were attached to culture dishes using silicone (Sylgard Dow Corning).

### Cell culture

The culturing procedures followed the ethical guidelines of Care and Use of Laboratory Animals and were approved by the Committee for Animal Experimentation at the Institute of Life Sciences of the Hebrew University. Primary cultures of CM were prepared as previously described (Shneyvays et al., 1998). The hearts of 1 day old rats were removed, washed in phosphate-buffered saline (PBS), minced to small fragments, agitated in a solution of proteolytic enzymes (RDB, Biological Institute, Ness-Ziona, Israel). The cells were then diluted 1:50 in Ca^2+^ and Mg^2+^-free PBS at 25°C. Several cycles of dissociated cardiac cell collection were performed lasting 10–15 min each. Dulbecco's modified Eagle's medium (DMEM) containing 10% horse serum (Biological Industries, Kibbutz Beit Haemek, Israel) was added to supernatant suspensions containing dissociated cells. The mixture was centrifuged at 300 g for 5 min. The supernatant phase was discarded, and the cells were suspended again. The suspension of the cells was diluted to 1×10^6^ cells/ml, which were plated on the laminin-coated (20 μg/ml) surfaces. The cultures were incubated in a humidified atmosphere of 5% CO_2_, 95% air at 37°C. Confluent monolayers exhibiting spontaneous contractions were developed in culture within 2–3 days. The growth medium was replaced after 24 h and then every 3 days.

### Electron microscopy

For both SEM and TEM analysis cells cultured on the gold spine substrate were fixed, dehydrated, and embedded in Agar 100 within the culturing dish as previously described (Spira et al., 2003). Briefly, CM were fixed by 3% glutaraldehyde in 0.1 M cacodilate buffer, pH 7.4. The cells were then washed with 0.1 M cacodilate buffer (Agar Scientific, Stansted, England). The cells were post-fixed in 1% osmium tetroxide (Next Chimica, Centurion, South Africa) and 0.8% K_3_Fe(CN)_6_. Dehydration was carried out through a series of ethanol solutions. For TEM the neurons were embedded in Agar 100 (Agar Scientific, Stansted, England). Then the glass substrate was etched using 30% hydrofluoric acid (for approximately 3 h). The Ti/Au layer was partially etched by diluted Au etcher (I_2_/KI/H_2_O) and diluted HF (1:40), leaving the gold spine structures intact. Thereafter, the agar block including the cells was re-embedded in Agar 100 in a flat mold. This doubly-embedded preparation was then thin-sectioned.

Measurements of cleft width from TEM images were done digitally using the image analysis program ImageJ (NIH, USA) as previously described (Fendyur et al., 2011). Each image was divided into three areas: (1) gold mushroom cap, (2) gold mushroom stalk, and (3) flat gold substrate in between the gold spines. The sampling locations were selected within a grid (100 nm pitch) randomly placed on the image. The distance between the cell membrane and the mushroom was measured along the corresponding fraction of the grid lines.

### Electrophysiology

For recording and electroporation gMμE-based MEA were used. In a series of preliminary experiments we selected the parameters for electroporation to be: positive square pulse duration of 50–100 ms and 0.5–1 V. The described experiments were then conducted in the following manner: we recorded spontaneous FP activity from the entire gMμE array for a number of minutes. We next selected gMμE that revealed FPs of ≥250 μV for electroporation. The amplitude of the applied electroporating pulse was gradually increased from 0.5 V in 100 mV steps until electroporation was monitored. Electroporation occurred in an all or none fashion. For electroporation of a number of CMs in the same culture dish we selected gMμEs that were at least 40 μm apart. After electroporation recordings lasted a number of minutes after the recovery of the electroporated membrane as evident by the reappearance of a typical extracellular potential. Altogether we have conducted >40 electroporating experiments using 30 cultures. For studies of repeated electroporation over days the cultures were kept in the incubator in between the experimental sessions. The signals were amplified by an AC, 60-channel amplifier (MEA-1060-Inv-BC, MCS) with frequency limits of 1–10,000 Hz at a sampling rate of 50 kHz. Origin 8.1 software (OriginLab Corp., Northampton, MA, USA) was used to plot the results.

## Results

### Structural relationships between the cardiomyocytes and the gold mushrooms microelectrode substrate

As the morphology and physiology of cultured rat CMs is influenced by the sub-micrometer topography and stiffness of the culturing substrate (for example Wang et al., 2011) we began the study by testing the compatibility of laminin coated gold-mushroom microstructures as a substrate for culturing dissociated rat CMs. To that end we compared the development of plated dissociated CMs (density of 1×10^6^ cells/ml) on flat glass surfaces and on matrices of 1–2 μm gMμEs fabricated on glass spattered by a thin film of gold. The inter-gMμEs interval was 8 μm. Both substrates were coated by laminin. On both substrates the cells adhered and began to spontaneously contract within 2–3 days after plating.

We next processed beating cultured CM for transmission and scanning electron microscopic examination (TEM and SEM respectively). Structural analysis (of 4–6 day old cultured CM) revealed two cell types: cardiac muscle cells characterized by a typical acto-myosin filament packing pattern, dark glycogen particles, and mitochondria and fibroblasts that lack acto-myosin machinery (Figure 1). Many of the CMs and fibroblasts adhered to the flat substrate in between the gMμE, and engulfed at least one gMμE (Figure 1). The averaged width of the extracellular cleft formed between adjacent CMs was in the range of 28.8 ± 2.1 (mean ± SE) nm [10 different CM-gMμE junctions were analyzed as previously described by us (Fendyur et al., 2011)]. Thirty-four percent of the CM-plasma membrane—gMμEs junctions appeared to be in direct contact (defined as 0–5 nm cleft). Twenty-two percent of the junctional area revealed a space of 5–25 nm, 18% of the junctional area ranged between 25 and 50 nm, 19% in the range of 50–100 nm, and 7% above 100 nm. The extracellular space formed between the myocytes and the flat substrate (in-between the electrodes) was 98 ± 5.7 (mean ± SE) nm (Figure 1). Since laminin was homogeneously applied to the culture substrate it is reasonable to conclude that the geometry of the gMμEs facilitated the formation of the reduced extracellular space between the gMμEs and the cell's membrane.

**Figure A1:**
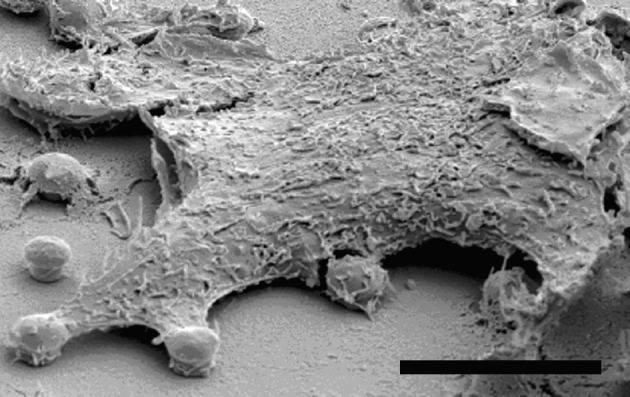
**A scanning electron micrograph depicting the relationships between a cultured cardiomyocyte and gMμEs.** Calibration: 10 μm.

Using SEM complemented by TEM observations we found that individual gMμEs were often engulfed by thin protrusions that extend from the cell bodies (Figure 1). In some cases the thin branches adhered to the mushroom cap and to parts of the “mushroom's stalk” leaving other parts of the gMμE stalk in contact with the extracellular solution (see also SEM image Figure A1).

### Extracellular field potential recordings by the gold mushroom-based MEA

The MEAs used in this study were composed of 8×8 gMμEs (two of which were not bonded) with a cap diameter of 1–2 μm and a pitch of 20 μm covering an area of 147×147 μm (~21,000 μm^2^). The gMμEs and the flat glass substrate in between the gMμEs were functionalized by laminin. Because of the small dimensions of the gMμEs and their pitch, a number of gMμEs were expected to be in physical contact with a single CM (with an estimated diameter of ~40 μm) and only a few CM could be recorded from. Recordings and electroporation pulses were made by 62 gMμEs using the Multi Channel Systems (Reutlingen, Germany) AC amplifier (MEA-1060-Inv-BC), with frequency limits of 1–10,000 Hz and a gain of 110–1100. The data shown is of raw, unprocessed recordings. Typically, the background noise level of the system was ~20 μV. In all experiments a 20 ms 1mV voltage calibration square pulse was applied to the bathing solution by an isolated pulse generator.

**Figure 2:**
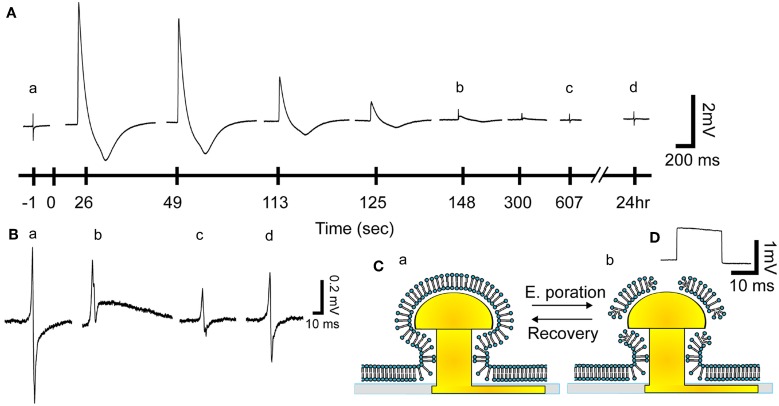
**From extracellular field potential (FP) recordings to intracellular recordings and the recovery process. (Aa)** Before electroporation a gMμE recorded a typical extracellular FP (enlarged in **Ba**). After the delivery of an electroporating pulse (100 ms 1 V) (at 0 s) the biphasic recorded FP transformed into a monophasic 5 mV positive potential with a shape similar to that reported by intracellular recordings. The amplitude of the action potential diminished over time, gradually resuming the shape of the extracellular field potential 125–148 s after electroporation. Thereafter the shape of the FP gradually recovered (between 148 and 607 s and enlarged in **B**) regaining the typical biphasic shape (**Ad** and **Bd**). **(B)** Enlargements of the FPs before electroporation **(Ba)** and **b** 148, **c** 607 s and **d** 24 h after electroporation. **(C)** Schematic drawing of the presumed reversible effects of an electroporating pulse on the plasma membrane facing a gMμE. **(D)** A calibration square pulse delivered to the bathing solution.

**Figure 3:**
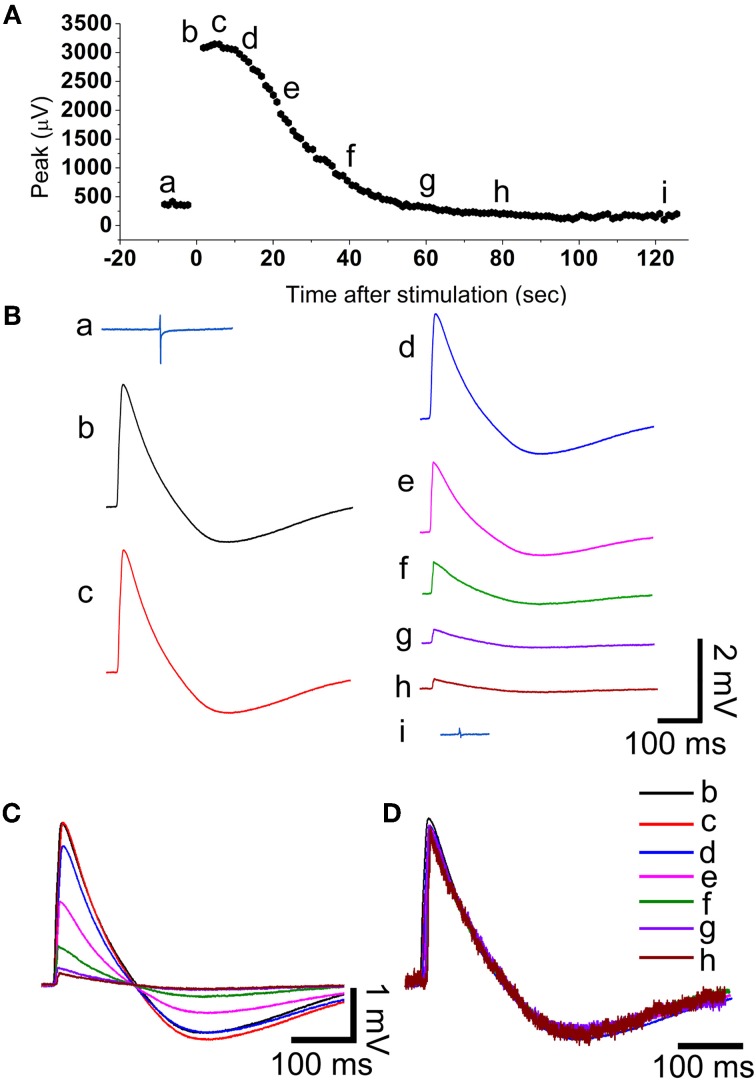
**During the recovery from electroporation the amplitude of the action potential is reduced but its shape is maintained. (A)** The amplitude of the filed potential (**Aa** and **Ba**) before electroporation and as a function of time after electroporation (**Ab–i** and **Bb–i**). The field potentials and action potentials shown in **B** are samples in time as indicated in **(A)**. **(C)** Super positioning of the action potentials shown in **(B)**. **(D)** The normalized action potentials shown in **(C)**. Note that the shape of the action potentials is almost identical suggesting that the decreased amplitude represents a gradual decrease in the membrane conductance facing the gold mushroom microelectrode.

**Figure 4:**
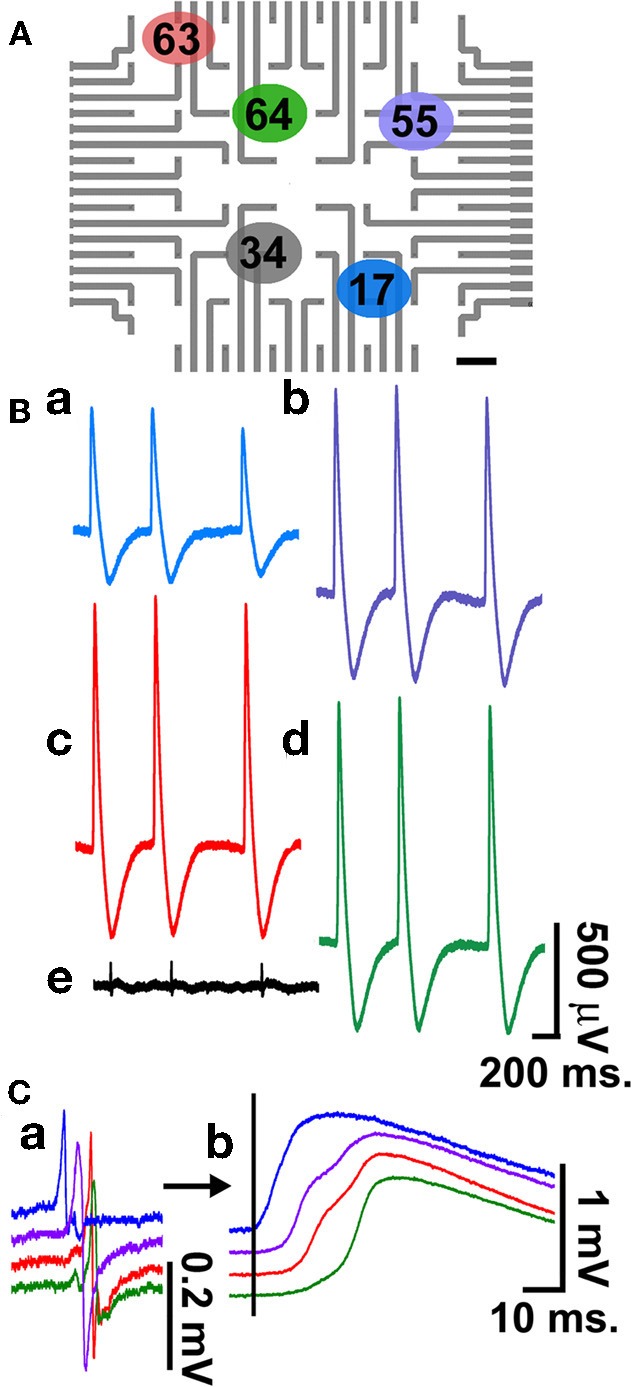
**Simultaneous multi-CM recordings.** Recordings of intracellular action potentials and FPs generated by electrically coupled, beating cultured CM grown on laminin functionalized gMμEs-based MEA for 4 days. For the experiment an electroporating pulse was delivered to electrodes 17(a), 55(b), 63(c), and 64(d), **(A)**. Recordings are shown from these electrodes and from electrode 34(e), which was not electroporated **(B)**. Superpositioning of the FPs prior to electroporation **(Ca)** and intracellular action potentials **(b)** reveals a 6–8 ms delay in the onset of the depolarization consistent with the propagation of the electrical impulse between the cells.

Recording of spontaneous electrical activity was made from 3 to 14 days old cultures. Altogether we recorded spontaneous field potentials from >50 cultures). Although the estimated surface area of a gMμE is small (~10 μm^2^) the FPs recorded by individual gMμE are very similar in amplitude and shapes to those reported earlier using larger substrate integrated planar electrodes (Banach et al., 2003; Reppel et al., 2005; Jones et al., 2011). This is consistent with the transmission electron micrographs indicating that the engulfment of the gMμEs by the cells increases the seal resistance formed between the cells and the electrode and thereby compensates for the relative smaller surface area of the electrodes (Figure 1; Hai et al., 2009a,b, 2010a,b; Fendyur et al., 2011). Most recorded FPs were composed of a positive phase representing the outward current generated by neighboring cells to the one from which the recording is made, followed by an inward phase that subsides to the baseline representing the upstroke phase of the AP and its repolarization to the resting level (Figures 2, 3 and 4; see Halbach et al., 2003).

### From extracellular to intracellular recordings

**Figure 5:**
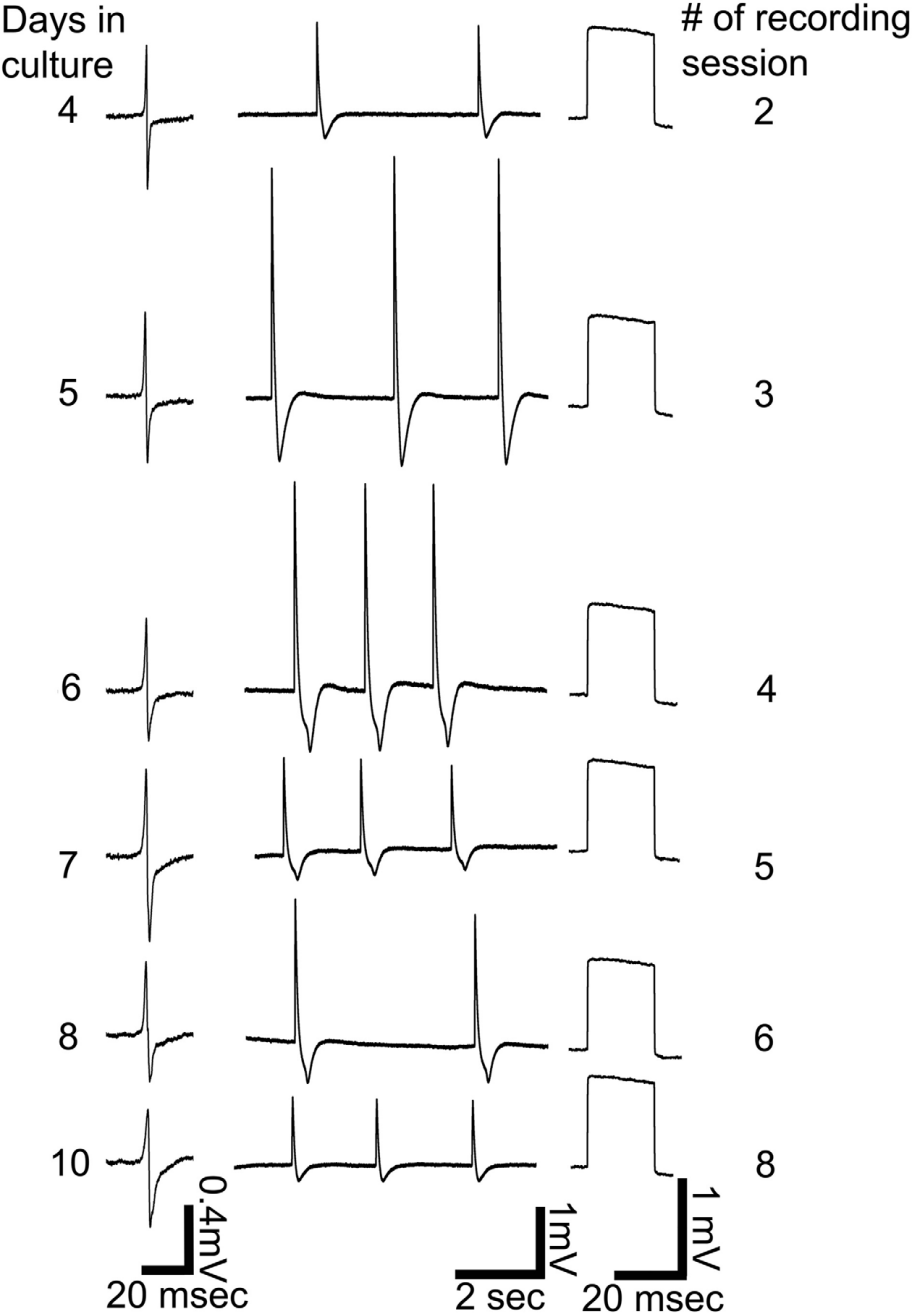
**Repeated intracellular recordings by a single gMμE on days 4, 5, 6, 7, 8, and 10 after plating.** Left column, extracellular field potentials before electroporation, middle column, spontaneous firing after electroporation, right column, calibration pulses of 1 mV 20 ms.

Application of a single 50–100 ms long 0.5–1 V positive square pulse through a gMμE leads to a transition from typical biphasic extracellular recordings of FPs to an intracellular recording mode of attenuated APs ranging between 1 and 6 mV (Figures 2, 3, 4, and 5, 30 cultures, >40 individual CMs). The recorded potentials have the characteristic shape of cultured rat CM APs with a sharp rise time of 5–7 ms (rise times were measured between 10 and 90% of the AP's peak value), a slower decay time and an after-hyperpolarizing potential of 0.4–1.5 mV(Halbach et al., 2003). The transition from extracellular to intracellular recording of APs is attributed to localized increased conductance of the plasma membrane facing the gMμE by electroporation (Figure 1A and see Xie et al., 2012).

The amplitude of the intracellularly recorded AP gradually diminishes over 2–10 min and reverts to extracellular FP recording mode (Figures 2, 3). The nature by which the reduction in the AP amplitude proceeds (Figure 3) suggests that it occurs by recovery of the electroporated nanopores at the junctional membrane (Figure 1A) and not because of pathological processes that reduce the driving forces of inward and outward ionic currents across the plasma membrane. In support of these conclusions we found that during a substantial phase from electroporation, the shape of the AP is maintained constant while its amplitude is diminished. This is illustrated in the experiment of Figure 3 in which normalized APs were superimposed revealing that the AP shape is not altered while the amplitude is reduced (Figures 3C,D). This behavior is consistent with a gradual reduction in the conductance of the electroporated patch of the plasma membrane that faces the electrode (schematically depicted in Figure 2C). If the attenuation of the APs had been the consequence of increase in the intracellular calcium and sodium concentrations the ensuing reduction in the driving forces of these ions, would have changed the shape of the APs. At late phases of the recovery process, when the amplitude of the intracellularly recorded AP is reduced to value of the FP (prior to electroporation) the shape of the recorded AP represent complex contributions of extracellular (FP) and intracellular (APs) currents (for example Figures 2Ab,Bb). The recovery rate was not correlated to the age of the cells in culture or the amplitude of the attenuated AP.

### Multisite intracellular recordings

Simultaneous recording of FPs from a number of cultured CM is often used to characterize the spreading of APs along electrically coupled CM and thereby to assess and pharmacologically manipulate the electrical coupling between the cells. Using the gMμE-based MEA such observations can be made by simultaneous intracellular recordings from a number of CMs as depicted in Figure 4. For the experiment, an electroporating pulse was simultaneously delivered to 4 gMμEs that recorded extracellular FPs (Figure 4). This led to the transformation of the recording configuration from an extracellular to an intracellular mode (Figures 4Ca,Cb). It is important to note that other gMμEs through which an electroporating pulse was not delivered maintained their extracellular recording mode (Figure 4Be). Super-positioning of the FPs and the APs recorded by the four different electrodes (Figures 4Ca,Cb, respectively) revealed clear delays in the onset of the potentials, reflecting the network properties of these cultured myocytes.

### Repeated electroporation and long-term recordings

Another important feature of the gMμE-based MEA is that cultured CM can be electroporated a number of times over a number of days. Figure 5 shows six out of eight consecutive extracellular and intracellular recordings made by the same gMμE (presumably from the same CM) over a period of 11 days.

## Discussion

In the present study we began to develop the use of gMμE-based arrays for simultaneous intracellular recording of APs from many beating CMs. We report that: (a) the physical junction formed between cultured rat CM and laminin coated, micrometer sized gMμEs is sufficient to enable extracellular recording of FPs generated by individual myocytes for weeks and (b), that in response to a short electroporation pulse delivered through the gMμEs, the CM-gMμE configuration is converted from an extracellular recording configuration to accessing intracellular recordings of attenuated APs. The shape and duration of the recorded APs is similar to those recorded intracellularly from rat CM. Autonomous recovery of the electropores generated at the plasma membrane facing the gMμEs revert the recording mode to extracellular. The gMμEs-based device enables repeated transition from the extracellular to the intracellular mode of recordings over a number of days despite the continuous beating of the CM.

### Structural relationships between cultured cardiomyocytes and gMμE

Earlier studies documented that cultured CM sense sub-micrometric substrate topographies and respond by morphological adaptation and cytoskeletal reorganization (for example Wang et al., 2011). Consistent with the earlier studies, we noted that the CM responded to the presence of the gMμEs by their engulfment. Further to that, we documented that 34% of the CM-plasma membrane—gMμEs junctions appeared to be in direct contact (defined as 0–5 nm cleft) with the gMμE. Interestingly, thin branches that extend from the cells also engulf the gMμE and form a junction of close membrane apposition. Assessment of the dimensions of the extracellular cleft formed between the CM plasma membrane and the gMμE from TEM micrographs must take into consideration the possibility that the chemical fixation, dehydration, and embedding might generate alterations in the intracellular osmotic pressure and thereby generate structural artifacts (Studer et al., 2008). Our analysis revealed that the narrowest junctions were consistently formed between the CM membrane and the gMμE cap rather than with the flat substrate in between the protruding gMμE. It is conceivable that the engulfment of the 3-dimensional structure, by the aid of actin and other submembrane cytoskeletal elements generates mechanical tension around the curving geometry of the gMμE and that this mechanical tension may underlie the formation of a narrow cleft in-between the plasma membrane the gMμE cap and stalk. Nevertheless, there exists a possibility that the tight apposition formed at this site may be the outcome of a fixation artifact. The well preserved subcellular organelles in the electron micrographs, suggest that the fixation, dehydration, and embedding procedures did not produce osmotic pressure artifacts. It is also important to note that the presence of electron translucent breaks within the embedding material, mainly at the curving junctions between the cells and the gMμE cap as well as the stalk (Figure 1), suggests that mechanical tension generated within the embedding polymer (Agar 100) lead to the detachment of the plasma membrane from the surface of the gMμE cap only during sectioning of the Agar block or the observations rather than at earlier stages of the procedures.

### From extracellular field potentials to action potentials by electroporation and back

As shown in the experiments of Figures 2–5, prior to electroporation the recorded FPs are composed of a positive phase representing outward current generated by neighboring myocytes followed by a negative phase which represent the voltage-dependent inward currents. The “pure” biphasic potentials recorded by individual gMμE is consistent with the structural relationships of an engulfed gMμEs by a single CM. This structural configuration leads to partial electrical “isolation” of one gMμE from the others. Comparison of the FPs recorded by different gMμEs revealed some variability in their shapes. This variability is attributed to differences in the seal resistances formed by individual cells and the electrode, and the level of electrical coupling of one cell to the others. A clear delay in the onset of the FPs recorded by the different electrodes reflects the conduction velocity of the APs along the electrically coupled CM network (Figure 4). After electroporation, which gain Ohmic access to the cell interior, the recorded APs last longer and are significantly larger than the extracellular FPs (which depict the first derivative of the AP voltage over time). The attenuation of the APs amplitude by the system is attributed to the relatively low seal resistance formed between the plasma membrane of the CM and the laminin coated gMμE device (Fromherz, 2006; Hai et al., 2010a,b). It is important to note that the shape and amplitude of intracellularly recorded signals by the gMμEs-MEA and the AC amplifier used are expected to differ from those recorded by the DC coupled intracellular electrode. The expected differences are related to the impedance generated by the ionic bilayer formed at the interface between the gMμE and the culture medium (Mortari et al., 2007) and the AC amplifier used. Since the parameters that represent individual cell-gMμE junction are not identical, the alterations in the signal shape and attenuation factor are expected to differ for individual gMμE. Nevertheless, these individual alterations can be corrected in future development of the system using the calibration pulse as a reference.

### Transient electroporation

With time after electroporation, the nanopores generated by the electroporating pulse recover (Kinosita and Tsong, 1977; Powell et al., 1986; Tsong, 1991; Freeman et al., 1994). This process is associated with gradual reduction in the amplitude of the APs yet, their shape is maintained quite constant for as long as their amplitude is larger than that of the FPs (Figures 2, 3). The transition between intracellular recordings of APs to that of extracellular FPs is smooth and continuous. As the resistance of the electroporated membrane increases the amplitude of the intracellular component decreases and that of the FP becomes more noticeable (Figure 2). These cascades of events suggest that focal CM electroporation by micrometer size gMμE provides transient Ohmic access to the cells interior and that the recovery process exclude the gMμE from the cell. Since the biphasic shape of the FP recovers it is conceivable to assume that the extracellular space between the CM membrane and the electrode is not dramatically altered. This conclusion is also supported by the observation that a single CM can be repeatedly penetrated over times. Future studies will be devoted to improve the seal resistance formed between cultured CM and gMμE by the functionalization of the gMμE with a membrane like lipid coating (Almquist and Melosh, 2010; Duan et al., 2012). This is expected to improve the duration and amplitude of the recorded APs. In future development of our approach it will be interesting to consider the benefits of being able to gain transient intracellular excess to CM rather than a “permanent penetration into the cells.” Long-term microelectrode penetration may eventually rupture the plasma membrane due to friction between the rigid electrodes and beating myocytes.

## Conclusions

In conclusion, we demonstrated that the gold mushroom-shaped-based MEA has the potential to serve as a convenient tool for repeated, simultaneous, intracellular recordings from an unlimited number of beating CMs. This study together with recent “proof of concept” demonstrations of multiunit, long-term, intracellular recordings from excitable cells by micro-(Hai et al., 2010a,b; Fendyur et al., 2011) and nano-MEAs (Tian et al., 2010; Duan et al., 2012; Robinson et al., 2012; Xie et al., 2012) opens up exciting opportunities for basic research and medical applications.

### Conflict of interest statement

The authors declare that the research was conducted in the absence of any commercial or financial relationships that could be construed as a potential conflict of interest.
